# Association between Alcohol Intake and Hemoglobin A1c in the Korean Adults: The 2011-2013 Korea National Health and Nutrition Examination Survey

**DOI:** 10.1371/journal.pone.0167210

**Published:** 2016-11-28

**Authors:** Jae Won Hong, Jung Hyun Noh, Dong-Jun Kim

**Affiliations:** Department of Internal Medicine, Ilsan-Paik Hospital, College of Medicine, Inje University, Koyang, Gyeonggi-do, Republic of Korea; McMaster University, CANADA

## Abstract

**Background:**

Although alcohol consumption is commonly encountered in clinical practice, few studies have investigated the clinical significance of alcohol intake on the use of the hemoglobin A1c (HbA1c) level.

**Objectives:**

This study was performed to investigate the association between alcohol intake and HbA1c level in the general population.

**Methods:**

Among the 24,594 participants who participated in the 2011–2013 Korea National Health and Nutrition Examination Survey (KNHANES), 12,923 participants were analyzed in this study. We excluded diabetic patients currently taking antidiabetes medication. We compared the HbA1c level and proportions of patients with an HbA1c level of ≥5.7%, ≥6.1%, and ≥6.5% according to the fasting plasma glucose (FPG) concentration range and the amount of alcohol intake. The average amounts of daily alcohol intake were categorized into three groups: 0 g/day, <30 g/day, ≥30 g/day.

**Results:**

The mean HbA1c level was 5.65%, and the mean FPG concentration was 95.3 mg/dl. The percentages of patients with an HbA1c level of ≥5.7%, ≥6.1%, and ≥6.5% were 42.6%, 13.4%, and 4.5%, respectively. The average amount of alcohol intake was 12.3 g/day. The percentages of subjects with alcohol intake 0, <30, and ≥ 30 g/day were 16.5%, 69.7%, and 13.8%, respectively.

There was a significant positive relationship between alcohol intake and FPG concentration (P < 0.001), the prevalence of impaired fasting glucose (P < 0.001), and the prevalence of diabetes (P < 0.001). However, there was no significant relationship between the alcohol intake and HbA1c level.

Overall, the adjusted HbA1c levels decreased across alcohol intake (5.70% ± 0.01%, 5.66% ± 0.01%, and 5.55% ± 0.01%) after adjustment for confounding factors such as age, sex, FPG concentration, college graduation, smoking history, presence of hypertension, waist circumference, serum total cholesterol concentration, serum high-density lipoprotein cholesterol concentration, serum triglyceride concentration, presence of anemia, serum white blood cell count, and serum alanine aminotransferase concentration (P < 0.001). The adjusted proportions (%) of patients with an HbA1c level of ≥5.7% (P < 0.001), ≥6.1% (P < 0.001), and ≥6.5% (P < 0.001) showed significant negative trends across alcohol intake after adjustment for confounders. Logistic regression analyses showed that, when using the group that abstained as the control, the group that consumed ≥ 30g/day was negatively associated with the risk of an HbA1c level of ≥5.7% (P < 0.001), ≥6.1% (P < 0.001), and ≥6.5% (P < 0.001), using the above-mentioned variables as covariates.

**Conclusions:**

Higher alcohol intake was associated with lower HbA1c levels, even after adjusting for confounding factors, including the FPG concentration, in this nationally representative sample of Korean adults. These results suggest that excessive drinking shifts the HbA1c level downward, which might complicate use of the HbA1c level for the diagnosis of diabetes or prediabetes.

## Introduction

Hemoglobin A1c (HbA1c) is clinically measured to estimate a patient’s overall glycemic exposure and risk for long-term complications. It is currently used to guide management and adjust therapy in patients with diabetes. Additionally, measurement of the HbA1c level is now recommended for the diagnosis of diabetes and screening of individuals at high risk of developing diabetes [1]. Compared with measurement of the fasting plasma glucose (FPG) concentration or performance of the oral glucose tolerance test (OGTT), advantages of the HbA1c assay include no need for fasting or timed samples and substantially less biologic variability and preanalytic instability. HbA1c is also relatively unaffected by acute changes in the glucose concentration, such as those induced by acute stress or illness [1]. However, measurement of the HbA1c level can produce either falsely high or low results independently of the patient’s glycemic status; such measurement results are influenced by various factors such as age, race, hematologic conditions that alter red blood cell turnover, and illness-related factors [2–5]. Recent studies have shown that lifestyle parameters, such smoking and alcohol consumption, are also associated with the HbA1c level in the general population [6–8].

Among these lifestyle parameters, alcohol drinking is one of the most common social culture for Korean adults. The mean amount of per person is 30.1g/day for men and 6.6 g/day for women, according to Korean statistical data [9]. Although alcohol drinking is commonly encountered matter in clinical practice, few studies have investigated the clinical significance of alcohol intake on the use of HbA1c.

In the current study, we performed a cross-sectional analysis to investigate the association between the alcohol intake and HbA1c levels in Korean adults based on data from the 2011–2013 Korean National Health and Nutrition Examination Survey (KNHANES).

## Methods

### Study population and data collection

This study was based on data from the 2011–2013 KNHANES, a cross-sectional and nationally representative survey conducted by the Korean Center for Disease Control for Health Statistics. The KNHANES has been conducted periodically since 1998 to assess the health and nutritional status of the civilian, noninstitutionalized population of Korea. Participants were selected using proportional allocation–systematic sampling with multistage stratification. A standardized interview was conducted in the homes of the participants to collect information on demographic variables, family history, medical history, medications used, and a variety of other health-related variables. The health interview included an established questionnaire to determine the demographic and socioeconomic characteristics of the subjects including age, education level, occupation, income, marital status, smoking habit, alcohol consumption, exercise, previous and current diseases, and family disease history.

The subjects were asked whether they exercised with an intensity that caused slight breathing difficulty and sweating. Those who exercised regularly at moderate intensity were asked about the frequency with which they exercised per week and the length of time per exercise session. Regular exercise was defined as five or more exercise sessions per week. Alcohol consumption was assessed by questioning the subjects about their drinking behavior, including the average amount of drinking and drinking frequency, during the month prior to the interview. A standard drink was defined as a single glass of liquor, wine, or the Korean traditional distrilled liquor (So-ju). One bottle of beer (355mL) was counted as 1.6 standard drinks. We considered the amount of alcohol per 1 standard drink as 10g, and the average amounts of daily alcohol intake were calculated and categorized into 3 groups: 0 g/day, <30 g/day, ≥30 g/day. An average consumption of 30g per day or more, a level of exposure associated with health risks, was considered as heavy alcohol drinking [10–14]. Diabetes was defined as a FPG concentration of ≥126 mg/dl (7.0 mmol/L), an HbA1c level of ≥6.5%, current use of antidiabetes medication, or a previous diagnosis of diabetes by a physician. Obesity was defined as a body mass index (BMI) of ≥25 kg/m^2^ according to the Asia-Pacific obesity classification [15].

Height and weight were obtained using standardized techniques and equipment. Height was measured to the nearest 0.1 cm using a portable stadiometer (Seriter, Bismarck, ND). Weight was measured to the nearest 0.1 kg using a Giant-150N calibrated balance-beam scale (Hana, Seoul, Korea). The BMI was calculated by dividing the weight by the square of the height (kg/m^2^). Systolic and diastolic blood pressure was measured by standard methods using a sphygmomanometer while the patient was seated. Three measurements were recorded for all subjects at 5-min intervals, and the average of the second and third measurements was used in the analysis.

### Laboratory methods

Blood samples were collected in the morning after fasting for at least 8 h. The total cholesterol, FPG, triglyceride (TG), and high-density lipoprotein (HDL) cholesterol concentrations were measured using a Hitachi Automatic Analyzer 7600 (Hitachi, Tokyo, Japan). The HbA1c level was measured using high-performance liquid chromatography (HLC-723G7; Tosoh, Tokyo, Japan). The hemoglobin (Hb) level was measured using the cyanide-free sodium lauryl sulfate Hb detection method. Anemia was defined in accordance with the WHO criteria: Hb of <13 g/dl in men and <12 g/dl in women [16].

### Ethics statement

This study was approved by the institutional review board of Ilsan Paik Hospital, Republic of Korea (IRB 2016-07-011). After approval of the study proposal, the KNHANES dataset was made available at the request of the investigator. Because the dataset did not include any personal information and participants’ consent had already been given for the KNHANES, our study was exempt from participant consent.

### Statistical analyses

Differences in age according to alcohol intake were evaluated by analysis of variance, and the percentage of females was evaluated by the chi square test. To compare the age- and sex-adjusted demographics and clinical characteristics according to alcohol intake, analysis of covariance and the Bonferroni *post hoc* test were used.

General linear models were used to assess the HbA1c level and proportion (%) of subjects with an HbA1c level of ≥5.7%, ≥6.1%, and ≥6.5% according to alcohol intake before (Model 1) and after (Models 2–4) adjustment for confounders. Model 2 was characterized by adjustment for age, sex, and the FPG concentration. Model 3 was characterized by adjustment for smoking history (never, past, or current), presence of anemia, serum white blood cell (WBC) count, as well as age, sex, and FPG concentration. In Model 4, college graduation, the presence of hypertension, waist circumference, serum total cholesterol, serum HDL cholesterol, serum TG, serum alanine aminotransferase (ALT), and variables in model 3 were adjusted for in the analysis. A logistic regression analysis was used to evaluate the odds ratios of alcohol intake for HbA1c levels of ≥5.7%, ≥6.1%, and ≥6.5% as covariates with age, sex, FPG concentration, college graduation, smoking history, presence of hypertension, waist circumference, serum total cholesterol concentration, serum HDL cholesterol concentration, serum TG concentration, presence of anemia, serum WBC count, and serum ALT concentration.

The HbA1c cutoff value of 6.1% was selected because a recent Korean study showed that this level was the optimal corresponding value for diagnosing diabetes, with the criteria of an FPG concentration of ≥7.0 mmol/L and/or a 2-h FPG concentration of ≥11.1 mmol/L on the 75-g OGTT (63.8% sensitivity and 88.1% specificity) [17]. An HbA1c threshold of 5.7% had reasonable sensitivity (48.6%) and specificity (65.7%) for identification of prediabetes [17].

All tests were two-sided, and P values of <0.05 indicated statistical significance. Statistical analyses were performed using SPSS software (ver. 21.0 for Windows; IBM Corp., Armonk, NY).

## Results

### Demographics and clinical characteristics of the study population

**Fig 1:**
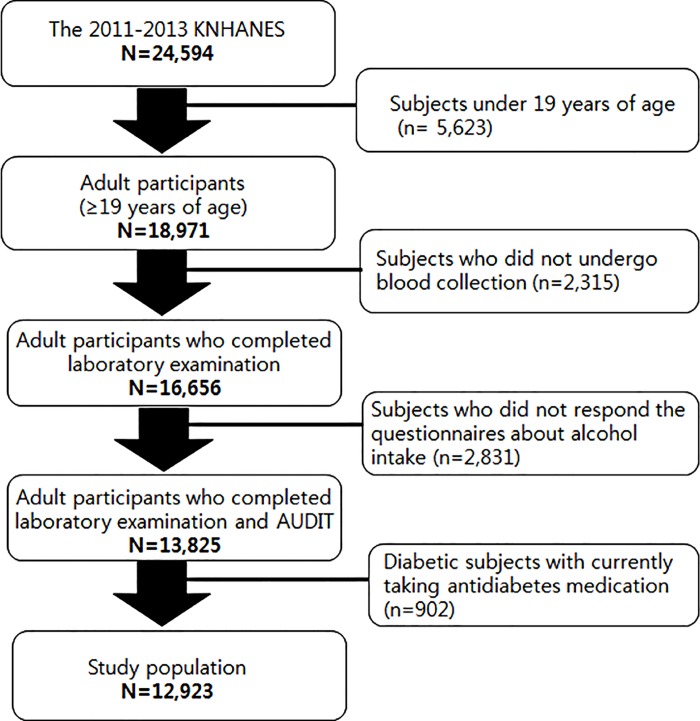
Participants disposition to be included in this study.

Among the 24,594 participants who participated in the 2011–2013 KNHANES, 5,623 subjects under 19 years of age were excluded. A total of 2,315 adults did not undergo blood collection and were excluded; 902 diabetic subjects currently taking antidiabetes medication and 2,831 subjects who did not respond the questionnaires about alcohol intake were also excluded. Finally 12,923 participants were analyzed in this study (Fig 1).

The demographics and clinical characteristics of the study population are shown in Table 1. The mean age was 46 years, and the percentage of females was 54%. The mean HbA1c level was 5.65%, and the mean FPG concentration was 95.3 mg/dl. The percentages of subjects with an FPG concentration of 100 to 125 mg/dl and ≥126 mg/dl were 6.4% and 3.2%, respectively. The percentages of subjects with an HbA1c level of ≥5.7%, ≥6.1%, and ≥6.5% were 42.6%, 13.4%, and 4.5%, respectively. The average amount of alcohol intake was 12.3 g/day. The percentages of subjects with alcohol intake 0, <30, and ≥ 30 g/day were 16.5%, 69.7%, and 13.8%, respectively.

**Table 1:** Demographic and clinical characteristics of Korean population without antidiabetes medication, aged 19 years and older, in 2011–2013 Korea National Health and Nutrition Examination Survey

Number	12,923
Age (year)	46 (19–92)
Women(%)	54.1
College graduation (%)	34.7
Alcohol intake (g/day)	12.3 ± 0.20
Alcohol intake (0,<30, ≥30g/day)%	16.5/69.7/13.9
Regular exercise (%)	7.9
Current smoking (%)	22.5
Waist circumference (cm)	80.6 ± 0.1
Body mass index (kg/m^2^)	23.6 ± 0.1
Obesity (%)	31.0
Systolic blood pressure (mmHg)	117.3 ± 0.1
Diastolic blood pressure (mmHg)	75.8 ± 0.1
Hypertension (%)	26.0
Serum total cholesterol (mg/dL)	189.7 ± 0.3
Serum triglyceride (mg/dL)	130.5 ± 0.9
Serum HDL-cholesterol (mg/dL)	53.1 ± 0.1
Fasting plasma glucose (mg/dL)	95.3 ± 0.1
HbA1c (%)	5.65 ± 0.01
HbA1c (mmmol/mol)	38.3 ± 0.11
100 ≤ FPG ≤ 125 mg/dL (%)	6.4
FPG ≥ 125 mg/dL (%)	3.2
HbA1c (%) ≥ 5.7 (%)	42.6
HbA1c (%) ≥ 6.1 (%)	13.4
HbA1c (%) ≥ 6.5 (%)	4.5
Serum hemoglobin (g/dL)	14.1 ± 0.1
Anemia (%)	7.8
Serum white blood cell counts (/μL)	5979 ± 15
Serum aspartate aminotransferase	22.1 ± 0.1
Serum alanine aminotransferase	21.3 ± 0.1

Data are expressed as mean with SEM. Regular exercise, ≥ ×5 exercise/week. Obesity, body mass index ≥ 25 kg/m^2^. Hypertension, systolic blood pressure ≥ 140 mmHg, diastolic blood pressure ≥ 90 mmHg, or current anti-hypertensive medication. Diabetes, fasting plasma glucose ≥ 126 mg/dL, or current anti-diabetes medication or a previous diagnosis of diabetes by a doctor. HDL, high-density lipoprotein. FPG, fasting plasma glucose, Anemia, serum hemoglobin < 13 g/dl in men and < 12 g/dl in women.

### Age, sex, and age- and sex-adjusted demographic and clinical characteristics according to alcohol intake

Table 2 shows the age- and sex-adjusted clinical characteristics according to alcohol intake (none, <30, and ≥ 30 g/day). The percentages of women and college graduates had a tendency to decrease along with the alcohol intake (P < 0.001). There was a significant positive relationship between the alcohol intake and FPG concentration (P < 0.001), the prevalence of impaired fasting glucose (P < 0.001), and the prevalence of diabetes (P < 0.001). However, there was no significant relationship between alcohol intake and HbA1c level. The systolic blood pressure (P < 0.001), diastolic blood pressure (P < 0.001), and prevalence of hypertension (P < 0.001) showed significant trends across the alcohol intake. The serum total cholesterol concentration (P < 0.001), serum HDL cholesterol concentration (P < 0.001), TG concentration (P < 0.001), and waist circumference (P < 0.001) increased with higher alcohol intake. The BMI (P < 0.001) and prevalence of obesity (P < 0.001) also increased along with alcohol intake. Current smoking (P < 0.001) was positively associated with alcohol intake. The serum aspartate aminotransferase (AST) concentration (P < 0.001), ALT concentration (P = 0.002) and serum WBC count (P<0.001) were higher in subjects with alcohol intake ≥ 30 g/day than in those with alcohol intake <30 g/day. There was a significant negative relationship between the percentage of anemia (P < 0.001) and alcohol intake.

**Table 2:** Age, sex, and age- and sex-adjusted demographic and clinical characteristics according to alcohol intake

Alcohol intake	None	<30g/day	≥30g/day	*P*
Number	2136	9010	1777	
The average amount of alcohol intake (g/day)	0	5.6 ± 0.1	61.1 ± 0.2	<0.001
Age (year)	53.4 ± 0.3	46.2 ± 0.2	45.6 ± 0.3	<0.001
Women(%)	68.8	58.2	15.2	<0.001
College graduation (%)	36.0 ± 1.0	35.4 ± 0.5	29.4 ± 1.1	<0.001
Regular exercise (%)	6.5 ± 0.3	8.1 ± 0.3	8.5 ± 0.7	0.029
Current smoking (%)	16.0 ± 0.8	20.3 ± 0.4	41.6 ± 10.9	<0.001
Waist circumference (cm)	80.0± 0.1	80.3 ± 0.1	82.5± 0.2	<0.001
Body mass index (kg/m^2^)	23.5 ± 0.1	23.6 ± 0.1	24.1 ± 0.1	<0.001
Obesity (%)	30.0 ± 0.1	29.8 ± 0.1	38.1 ± 0.1	<0.001
Systolic BP (mmHg)	116.2 ± 0.3	116.7 ± 0.2	121.4 ± 0.4	<0.001
Diastolic BP (mmHg)	74.5 ± 0.2	75.4 ± 0.1	79.1 ± 0.3	<0.001
Hypertension (%)	24.2 ± 0.9	24.6 ± 0.4	35.5 ± 1.0	<0.001
Total cholesterol (mg/dL)	188.3 ± 0.8	189.3 ± 0.4	193.7 ± 0.9	<0.001
Serum triglyceride (mg/dL)	124.2 ± 2.3	124.3 ± 1.2	169.2 ± 2.5	<0.001
HDL-cholesterol (mg/dL)	50.3 ± 0.3	52.9 ± 0.1	57.4 ± 0.3	<0.001
FPG (mg/dL)	93.7 ± 0.4	95.0 ± 0.2	98.8 ± 0.4	<0.001
HbA1c (%)	5.67 ± 0.01	5.65 ± 0.01	5.65 ± 0.01	0.607
HbA1c (mmmol/mol)	38.5 ± 0.11	38.3 ± 0.11	38.3 ± 0.11	0.607
100 ≤ FPG ≤ 125 mg/dL (%)	5.4 ± 0.5	6.0 ± 0.3	9.9 ± 0.6	<0.001
FPG ≥ 125 mg/dL (%)	2.7 ± 0.4	2.9 ± 0.2	5.1 ± 0.4	<0.001
Serum hemoglobin (g/dL)	14.0 ± 0.1	14.1 ± 0.1	14.3 ± 0.1	<0.001
Anemia (%)	10.3 ± 0.6	7.6 ± 0.3	5.8 ± 0.7	<0.001
Serum WBC counts (/μL)	6038 ± 37	5940 ± 17	6099 ± 41	<0.001
Serum AST	21.4 ± 0.3	21.6 ± 0.1	25.5 ± 0.3	<0.001
Serum ALT	21.7 ± 0.4	21.0 ± 0.2	22.7 ± 0.5	0.002

Data are expressed as mean with SEM. Regular exercise, ≥ ×5 exercise/week. Obesity, body mass index ≥ 25 kg/m^2^. BP, blood pressure. Hypertension, systolic blood pressure ≥ 140 mmHg, diastolic blood pressure ≥ 90 mmHg, or current anti-hypertensive medication. Diabetes, fasting plasma glucose ≥ 126 mg/dL, or current anti-diabetes medication or a previous diagnosis of diabetes by a doctor. HDL, high-density lipoprotein. FPG, fasting plasma glucose, Anemia, serum hemoglobin < 13 g/dl in men and < 12 g/dl in women. WBC, white blood cell. AST, aspartate aminotransferase. ATL, alanine aminotransferase.

### Adjusted HbA1c level according to alcohol intake

We assessed the adjusted HbA1c levels according to alcohol intake and the range of FPG concentrations (Table 3). Overall, the adjusted HbA1c levels decreased across alcohol intake (5.70% ± 0.01%, 5.66% ± 0.01%, and 5.55% ± 0.01%) after adjustment for confounding factors (Models 4).

**Table 3:** Adjusted HbA1c (%) according to alcohol intake

Alcohol intake (g/day)	None	<30 g/day	≥ 30 g/day	*P*
**Total**				
Number	8831	2665	1427	
Model 1	5.73 ± 0.01	5.64 ± 0.01	5.65 ± 0.02	<0.001
Model 2	5.71 ± 0.01	5.66 ± 0.01	5.55 ± 0.01	<0.001
Model 3	5.71 ± 0.01	5.67 ± 0.01	5.54 ± 0.01	<0.001
Model 4	5.70 ± 0.01	5.66 ± 0.01	5.55 ± 0.01	<0.001
FPG <100 mg/dL				
Number	1929	8258	1495	
Model 1	5.64 ± 0.01	5.55 ± 0.01	5.51 ± 0.01	<0.001
Model 2	5.60 ± 0.01	5.56 ± 0.01	5.49 ± 0.01	<0.001
Model 3	5.60 ± 0.01	5.56 ± 0.01	5.48 ± 0.01	<0.001
Model 4	5.60 ± 0.01	5.56 ± 0.01	5.49 ± 0.01	<0.001
100≤ FPG <126 mg/dL				
Number	141	506	185	
Model 1	6.22 ± 0.04	6.12 ± 0.02	5.94 ± 0.04	<0.001
Model 2	6.17 ± 0.04	6.13 ± 0.02	5.96 ± 0.04	<0.001
Model 3	6.17 ± 0.04	6.13 ± 0.02	5.95 ± 0.04	<0.001
Model 4	6.14 ± 0.04	6.13 ± 0.02	5.97 ± 0.04	0.006
FPG ≥ 126 mg/dL				
Number	66	246	97	
Model 1	7.16 ± 0.21	7.64 ± 0.11	7.28 ± 0.17	0.052
Model 2	7.52 ± 0.10	7.56 ± 0.05	7.22 ± 0.09	0.004
Model 3	7.50 ± 0.10	7.56 ± 0.05	7.24 ± 0.09	0.010
Model 4	7.45 ± 0.10	7.55 ± 0.05	7.30 ± 0.09	0.057

FPG, fasting plasma glucose.

Model 1: unadjusted

Model 2: adjusted for age, sex, and fasting plasma glucose

Model 3: adjusted for smoking history (never, past, and current smoking), the presence of anemia, serum white blood cell counts, and variables in model 2

Model 4: adjusted for college graduation, the presence of hypertension, waist circumference, serum total cholesterol, serum high-density lipoprotein-cholesterol, serum triglyceride, serum alanine aminotransferase, and variables in model 3

Among subjects with an FPG concentration of <100 mg/dl, the HbA1c level decreased along with alcohol intake (P < 0.001) before (Model 1) and after (Models 2–4) adjustment for confounders. Among subjects with an FPG concentration of 100 to 125 mg/dl, the adjusted HbA1c level also decreased along with alcohol intake before (Model 1) and after (Models 2–4) adjustment for confounders. Among subjects with an FPG concentration of ≥126 mg/d, the HbA1c levels showed a tendency to decrease according to alcohol intake after adjustment for confounding factors (Models 2 and 3). However, there were no statistical significance between the HbA1c level and alcohol intake in Model 1 (P = 0.052) and Model 4 (P = 0.057).

### Adjusted proportions (%) of HbA1c levels of ≥5.7%, ≥6.1%, and ≥6.5% according to alcohol intake

The proportions (%) of HbA1c levels of ≥5.7%, ≥6.1%, and ≥6.5% according to alcohol intake are shown in Table 4. The unadjusted and adjusted proportions (%) of an HbA1c level of ≥5.7%, ≥6.1%, and ≥6.5% were also negatively associated with alcohol intake (Model 1–4, P<0.001). Spearman’s correlation coefficient between HbA1c and alcohol intake (g/day) were -0.093, -0.122, -0.133 and -0.120 in Model 1–4, respectively (P<0.001).

**Table 4:** Adjusted proportion of HbA1c ≥ 5.7%, ≥ 6.1%, and ≥ 6.5% (%) according to alcohol intake

Alcohol intake (g/day)	0		<30		≥30	*P*
HbA1c (%) ≥ 5.7(%)						
Model 1		50.5 ± 1.1		41.3 ± 0.5	39.4 ± 1.2	<0.001
Model 2		45.6 ± 1.0		43.0 ± 0.5	36.8 ± 1.1	<0.001
Model 3		45.2 ± 1.0		43.2 ± 0.5	36.2 ± 1.1	<0.001
Model 4		44.8 ± 1.0		43.3 ± 0.5	36.2 ± 1.1	<0.001
HbA1c (%) ≥ 6.1(%)						
Model 1		18.4 ± 0.7		12.5 ± 0.4	12.1 ± 0.8	<0.001
Model 2		16.5 ± 0.6		13.5 ± 0.3	9.4 ± 0.7	<0.001
Model 3		16.4 ± 0.6		13.6 ± 0.3	9.0 ± 0.7	<0.001
Model 4		16.2 ± 0.6		13.6 ± 0.3	8.9 ± 0.8	<0.001
HbA1c (%) ≥ 6.5(%)						
Model 1		6.0 ± 0.4		4.1 ± 0.2	4.6 ± 0.5	<0.001
Model 2		6.1 ± 0.4		4.6 ± 0.2	2.1 ± 0.4	<0.001
Model 3		6.1 ± 0.4		4.6 ± 0.2	1.9 ± 0.4	<0.001
Model 4		6.1 ± 0.4		4.6 ± 0.2	1.9 ± 0.4	<0.001

Model 1: unadjusted

Model 2: adjusted for age, sex, and fasting plasma glucose

Model 3: adjusted for smoking history (never, past, and current smoking), the presence of anemia, serum white blood cell counts, and variables in model 2

Model 4: adjusted for college graduation, the presence of hypertension, waist circumference, serum total cholesterol, serum high-density lipoprotein-cholesterol, serum triglyceride, serum alanine aminotransferase, and variables in model 3

### Odds ratios of alcohol intake to predict the risks of HbA1c levels of ≥5.7%, ≥6.1%, and ≥6.5%

We performed logistic regression analyses of alcohol intake with respect to the risk of an HbA1c level of ≥5.7%, ≥6.1%, and ≥6.5% using age, sex, FPG concentration, college graduation, smoking history, presence of hypertension, waist circumference, serum total cholesterol concentration, serum HDL cholesterol concentration, serum TG concentration, presence of anemia, serum WBC count, and serum ALT concentration as covariates. The odds ratios of alcohol intake to predict the risk of an HbA1c level of ≥5.7%, ≥6.1%, and ≥6.5% are shown in Table 5. Using the group that abstained as the control, the group that consumed ≥ 30g/day was negatively associated with HbA1c levels of ≥5.7%, the current cutoff value for abnormal glucose regulation. For HbA1c levels of ≥6.1%, using the group that abstained as the control, the group that consumed ≥ 30g/day and <30g/day decreased the risk by 58.0% (P < 0.001) and 17.0% (P < 0.001), respectively. For HbA1c levels of ≥6.5%, using the group that abstained as the control, the odds ratios of the group that consumed ≥ 30g/day was 0.32 (95% CI, 0.20–0.52; P < 0.001). A negative association between alcohol intake ≥ 30g/day and the risk of an HbA1c level of ≥5.7%, ≥6.1% and ≥6.5% was found in both men and women. However, the group that consumed < 30g/day decreased the risk of HbA1c ≥6.1% only in men, using the group that abstained as the control (95% CI, 0.50–0.84; P = 0.001).

**Table 5:** Odds ratios (ORs) of alcohol intake to predict the risk of HbA1c levels of ≥ 5.7%, ≥ 6.1%, and ≥ 6.5%

	For HbA1c ≥ 5.7%		For HbA1c ≥ 6.1%		For HbA1c ≥ 6.5%	
	OR (95% CI)	*P*	OR (95% CI)	*P*	OR (95% CI)	*P*
**Total**						
None	Reference		Reference		Reference	
Alcohol intake (<30 g/day)	0.90 (0.80–1.01)	0.060	0.83 (0.70–0.97)	0.022	0.82 (0.61–1.09)	0.175
Alcohol intake (≥30 g/day)	0.56 (0.47–0.66)	<0.001	0.42 (0.32–0.54)	<0.001	0.32 (0.20–0.52)	<0.001
**Men**						
None	Reference		Reference		Reference	
Alcohol intake (<30 g/day)	0.85 (0.70–1.04)	0.106	0.65 (0.50–0.84)	0.001	0.64 (0.41–1.00)	0.050
Alcohol intake (≥30 g/day)	0.56 (0.44–0.70)	<0.001	0.40 (0.29–0.56)	<0.001	0.32 (0.18–0.57)	<0.001
**Women**						
None	Reference		Reference		Reference	
Alcohol intake (<30 g/day)	0.91 (0.79–1.05)	0.211	0.98 (0.79–1.21)	0.856	0.97 (0.66–1.41)	0.855
Alcohol intake (≥30 g/day)	0.37 (0.25–0.55)	<0.001	0.22 (0.10–0.45)	<0.001	0.12 (0.02–0.59)	0.009

Covariates: age, sex, and fasting plasma glucose, college graduation, smoking history (never, past, and current smoking), the presence of hypertension, waist circumference, serum total cholesterol, serum high-density lipoprotein-cholesterol, serum triglyceride, the presence of anemia, serum white blood cell counts, serum alanine aminotransferase.

## Discussion

Using the KNHANES 2011–2012 data, we found that heavy alcohol consumption decreased the HbA1c level independently of the FPG concentration in the general adult population of Korea, even after adjustment for several confounding factors. This change occurred both in the normoglycemic range with an FPG concentration of <100 mg/dl and the prediabetic range with an FPG concentration of 100 to 125 mg/dl.

Previous epidemiological studies have focused on the association between alcohol intake and the risk of diabetes. Although heavy alcohol consumption is often regarded as a risk factor for diabetes, moderate alcohol consumption is known to be associated with a decreased incidence of diabetes [18,19]. Several meta-analyses have shown evidence of a considerably reduced risk of type 2 diabetes associated with moderate but not heavy alcohol consumption in men and women, indicating a U-shaped relationship [20,21]. In the present study, higher alcohol drinking showed a significant positive association with the FPG concentration, prevalence of impaired fasting glucose, and prevalence of diabetes. However, there was no association between the HbA1c level and alcohol intake, suggesting that alcohol intake might have a negative influence on the HbA1c level regardless of the individual’s glycemic status.

A few studies have shown the effect of alcohol intake on the HbA1c level in general populations without diabetes. In the European Prospective Investigation into Cancer (EPIC)-Norfolk study, a population-based cohort study of diet and chronic disease, alcohol intake was associated with a lower HbA1c level in nondiabetic men and women [22]. Gulliford *et al*. showed that the glycated Hb concentration was 0.189% lower in those who drank ≥42 units of alcohol per week than in nondrinkers among nondiabetic European subjects [6]. However, these studies suggest that consumption of alcohol leads to improved insulin sensitivity with lower blood glucose concentrations, leading to lower HbA1c levels.

Unlike previous studies, we adjusted for the FPG concentration and demonstrated the effect of alcohol intake as a nonglycemic determinant of the HbA1c level. Previously, Jansen *et al*. reported that alcohol consumption was associated with the HbA1c level independently of glycemia, which was similar to our result [7].

We also found that a heavy alcohol consumption affected the HbA1c distribution with lower proportions of HbA1c levels of ≥5.7% and ≥6.5%. This result suggests that excessive drinking shifts the HbA1c level downward, even after adjusting for confounding factors, including the FPG concentration. This might complicate the use of the HbA1c level for the diagnosis of diabetes or prediabetes. Therefore, clinicians should consider measuring the FPG concentration or performing the OGTT for diagnosis of diabetes or prediabetes in heavy drinkers whose HbA1c levels are close to the diagnostic threshold.

The mechanisms leading to decreased HbA1c levels in patients with higher alcohol intake remain unclear. First, we consider that alcohol affects glycation or certain assays, regardless of glucose-mediated effects, because this association was present between alcohol intake and the HbA1c level, independently of the FPG concentration. There has been conflicting evidence concerning the effect of acetaldehyde, a reactive metabolite of alcohol, on the HbA1c level. Steven *et al*. reported that alcoholics were found to have subnormal amounts of HbA1c, as determined by radioimmunoassay [23]. However, elevated HbA1c levels in alcoholics, measured by chromatography, have been detected in other studies [24,25]. Meanwhile, Koskinen *et al*. showed that the *in vivo* effects of alcohol are negligible in routine HbA1 measurement [26].

Second, although we performed our analyses after adjustment for the FPG concentration, a residual or hidden effect of the blood glucose level on the association between the HbA1c level and alcohol intake cannot be excluded. Improved insulin sensitivity by alcohol intake, resulting in a lower blood glucose concentration and HbA1c level, has been suggested to play a role in this association [6,22].

Third, we speculate that factors related to heavy alcohol intake might account for the altered HbA1c levels. Malnutrition, a shortened erythrocyte lifespan, and chronic liver disease even in the absence of cirrhosis and splenomegaly in heavy alcohol drinkers could contribute to decreased HbA1c levels in subjects with heavy drinkers [2,26].

There are several strengths to this study. First, we examined a large, nationally representative sample of adult Koreans. Second, patients with diabetes not currently taking antidiabetes medication were included in this study, allowing us to assess the impact of alcohol drinking on the HbA1c distribution at higher HbA1c levels (i.e., <6.5% vs. ≥6.5%, the diagnostic cutoff point for diabetes recommended by the American Diabetes Association). Although the impact of a heavy alcohol consumption on the HbA1c level was relatively small, this is the first large-population study to investigate the clinical effect of alcohol intake on the use of the HbA1c level.

Nevertheless, this study also had some limitations. First, this was not a prospective observational study, and its cross-sectional design prevented us from drawing conclusions regarding causality. Second, we categorized the subjects into three alcohol-drinking groups based on a self-reported questionnaire. This could lead to misclassification of the actual drinking pattern because participants could underestimate their alcohol consumption by recall error or intentionally. Finally, there are variations in acetaldehyde metabolism via genetically determined polymorphisms in aldehyde dehydrogenases (ALDH) enzymes [27]. The ALDH polymorphism seems to play an important role in ethnic differences on the systemic effect of alcohol drinking as well as vulnerability to organ damage [28]. Therefore, the effect of alcohol intake on the HbA1c level might be different in other ethnic group.

In conclusion, higher alcohol consumption was associated with lower HbA1c level, even after adjusting for confounding factors, including the FPG concentration, in this nationally representative sample of Korean adults. These results suggest that excessive drinking shifts the HbA1c level downward, which might complicate use of the HbA1c level for the diagnosis of diabetes or prediabetes.
